# Case of Acute Peritoneal Enteritis

**Published:** 1840-05-16

**Authors:** William Zollickoffer

**Affiliations:** Middleburg, (Md.)


### CASE OF ACUTE PERITONEAL ENTERITIS.

BY WILLIAM ZOLLICKOFFER, M. D.

To the Editors of the Medical Examiner.

*Gentlemen*,—On the 2d of February, I was called to visit the wife of Mr. S———, of this place, who, I concluded, after minute examination, to be labouring under acute peritoneo-muscular inflammation of the bowels, the symptoms being such as to characterize, with considerable accuracy, this state of enteritic diseased action; with the exception of the condition of the pulse, in conjunction with the whole of the cutaneous surface, the latter not having undergone any modification in its aspect or temperature, nor the former being increased in either quickness or frequency beyond the natural standard, although the wide spread organic derangement throughout the whole intestinal canal, as exhibited from the *post mortem* examination twenty-four hours after her dissolution, was such as rendered it somewhat surprising that the surface and sanguiferous system, should have remained silent in expressing, to some extent, the morbid lesion of the parts involved in the case of Mrs. S. From what will be seen in the sequel of this paper, the present case will readily be acknowledged as a striking evidence illustrative of the difficulty under which the professional man sometimes labours, in forming a correct idea in relation to the extent of diseased action with which he has to combat, from the circumstance of some intervening unknown cause, preventing the general system from expressing all those symptoms by which it may clearly be ascertained.

In the present case, the bowels were obstinately constipated; so much so, that I was unable to procure a single dejection of any kind. The pain in the umbilical region was lancinating and incessant; the abdomen considerably tumefied and sensitive, and the pain increased upon the slightest pressure; tongue covered with a white fur, the edges of which were of a pale red; thirst very considerable. The third day singultus occurred. Fourth day stercoraceous vomiting commenced, and continued until the sixth day, when death closed the scene of Mrs. S.'s suffering.

The therapeutic course adopted in the treatment of this case was such as the urgency of the symptoms loudly called for,—venesection carried to a sufficient extent to produce an impression on the sanguiferous system. Cathartics and accoproctics of different kinds were varied to suit circumstances. Injections were frequently exhibited, from the emollient to the more stimulating, and epistastics applied over the whole extent of the abdomen, &c.

*Post Mortem appearances twenty-four hours after Death.*

The whole of the peritoneal and muscular coats of the bowels exhibited, from the pyloric orifice to the lower end of the rectum, a perfect mass of inflammation. The cœcum was mortified. There was a stricture in the ileum of three inches in length. Adhesion of a portion of the cœcum to the left side; also of the rectum throughout its course, to the left side of the sacrum. The peritoneal coat of the whole abdominal parietes exhibited the same state of inflammation as that of the intestinal canal; the bowels were enormously distended with flatus; mucous coat not impaired; stomach distended with the contents proper to the bowels; gall bladder filled with a viscid, dark fluid; liver rather lighter coloured than usual; the abdominal cavity contained a considerable quantity of a sero-purulent fluid. From the above representation of facts in relation to the case of Mrs. S———, it may justly be concluded that her disease terminated in effusion and gangrene.

In the above examination I was assisted by my esteemed and intelligent friend, James L. Billingsba, M. D. This communication has been drawn up in the most concentrated possible form, in order to cause it to occupy as little space as practicable, without, at the same time, omitting any thing of material importance. If it is deemed worth an insertion in the "Medical Examiner," you are at perfect liberty to give it publicity.

*Middleburg, (Md.) 7th May,* 1840.

